# ‘Free’ inhibin α subunit is expressed by bovine ovarian theca cells and its knockdown suppresses androgen production

**DOI:** 10.1038/s41598-019-55829-w

**Published:** 2019-12-24

**Authors:** Mhairi Laird, Claire Glister, Warakorn Cheewasopit, Leanne S. Satchell, Andrew B. Bicknell, Phil G. Knight

**Affiliations:** 1 School of Biological Sciences, Hopkins Building, University of Reading, Whiteknights, Reading UK; 2 Present Address: Department of Biology, Ramkhamhaeng University, Bangkapi, Bangkok Thailand

**Keywords:** Reverse transcription polymerase chain reaction, Endocrine reproductive disorders

## Abstract

Inhibins are ovarian dimeric glycoprotein hormones that suppress pituitary FSH production. They are synthesised by follicular granulosa cells as α plus βA/βB subunits (encoded by *INHA*, *INHBA*, *INHBB*, respectively). Inhibin concentrations are high in follicular fluid (FF) which is also abundant in ‘free’ α subunit, presumed to be of granulosal origin, but its role(s) remains obscure. Here, we report the unexpected finding that bovine theca cells show abundant INHA expression and ‘free’ inhibin α production. Thus, theca cells may contribute significantly to the inhibin α content of FF and peripheral blood. *In vitro*, knockdown of thecal *INHA* inhibited *INSL3* and *CYP17A1* expression and androgen production while *INSL3* knockdown reduced *INHA* and inhibin α secretion. These findings suggest a positive role of thecal inhibin α on androgen production. However, exogenous inhibin α did not raise androgen production. We hypothesised that inhibin α may modulate the opposing effects of BMP and inhibin on androgen production. However, this was not supported experimentally. Furthermore, neither circulating nor intrafollicular androgen concentrations differed between control and inhibin α-immunized heifers, casting further doubt on thecal inhibin α subunit having a significant role in modulating androgen production. Role(s), if any, played by thecal inhibin α remain elusive.

## Introduction

Inhibins are glycoproteins of gonadal origin that play a key role in the negative feedback regulation of FSH production by pituitary gonadotrophs. They were first isolated from ovarian follicular fluid in the mid-1980s and characterized as disulphide-linked heterodimeric proteins comprising an α subunit linked to one of two β subunits (βA or βB) (reviews^1–4^). The α, βA and βB subunit precursors are encoded by *INHA*, *INHBA* and *INHBB* genes, respectively; these are co-expressed primarily in ovarian granulosa cells^3,5^. Post-translational processing of the subunit precursors gives rise to mature inhibin forms (~32 kDa) as well as higher molecular mass (M*r*) forms, all of which appear to display inhibin-like biological activity (i.e. ability to suppress pituitary FSH secretion)^6–8^. In addition, βA and βB subunits form disulphide-linked homo- (βAβA, βBbB) or hetero-dimers (βAβB) referred to as activin A, activin B and activin AB, respectively^2,3,5^. Like inhibins, activins were also purified from in the mid 1980s but on the basis of their ability to enhance, rather than suppress, pituitary FSH secretion^2,4^. Inhibins oppose the actions of activins by competing for binding to type 2 activin receptors on the cell surface, an obligatory step in activin-induced signalling^3,9^.

As well as dimeric inhibins and activins, ‘free’ inhibin α subunit has previously been detected in, and isolated from, ovarian follicular fluid^10–12^. Proteolytic processing of full length inhibin α subunit precursor (pro-αNαC; MW~50 kDa) generates a series of lower molecular weight peptides including αNαC (MW ~43 kDa), pro-αC (~26 kDa) and αN (~23 kDa) but there have been relatively few studies to explore the functional significance of ‘free’ α subunit(s) as an intra-ovarian and/or peripheral regulator of cellular function. In contrast to dimeric inhibins, inhibin α subunit forms do not suppress the synthesis and secretion of FSH by pituitary gonadotrophs^10,11^. Nonetheless, several lines of evidence suggest that inhibin α subunit may have intrinsic biological activities distinct from dimeric inhibins and may serve as additional local modulators of follicular function. For instance, inhibin α subunit has been shown to inhibit the binding of FSH to granulosal FSH receptors^13^ and to modulate oocyte maturation in a bovine *in vitro* fertilization/embryo culture model^14^. Mice with targeted deletions of *Inha* develop ovarian tumours^15^ while a spontaneous missense mutation in the human *INHA* gene is associated with premature ovarian insufficiency and primary amenorrhea in women^16^.

In addition, evidence indicates that free inhibin α subunit may function as an inhibin antagonist (hence activin agonist) by competing with inhibin for binding to its co-receptor, betaglycan, to which inhibin binds via its α subunit^17,18^. Association with betaglycan on the cell surface greatly enhances the presentation of inhibin to type 2 activin receptors^18^. By reducing the amount of betaglycan available to bind inhibin, inhibin α subunit should compromise the interaction of inhibin with the type 2 activin receptor thereby leaving the receptor more freely accessible to activin. Betaglycan is expressed by bovine theca cells and levels increase with follicle development^19^. Inhibin has also been shown to antagonize BMP signalling in several cell-types including human hepatocytes^20^, mouse adrenocortical cells^21^, rat gonadotrophs^22^ and bovine theca cells^19^, reflecting the requirement for BMP interaction with type 2 activin/BMP receptors. It is proposed, therefore, that free inhibin α subunit may also function as a local enhancer of BMP signalling at the ovarian level.

Since ovarian granulosa cells are recognised as the principle site of inhibin/activin subunit mRNA expression (*INHA*, *INHBA* and *INHBB*) in various mammalian species it has logically been deduced that the high concentrations of dimeric inhibin (αβ dimer) and activin (ββ dimer) proteins present in follicular fluid are primarily of granulosal origin. Likewise, the high concentrations of free inhibin α subunit identified in follicular fluid were presumed to be mainly from granulosa cells^10–12^. In the present study we report evidence that challenges the latter assumption and raises the possibilty that free inhibin α subunit of thecal origin may have a local role to modulate follicular androgen production.

Herein, we first report the unexpected finding of high expression levels of *INHA* mRNA in bovine theca interna cells. This finding prompted a follow-on series of experiments to (a) confirm the presence of inhibin α subunit protein in theca cells and examine the cellular origin of inhibin α subunit found in bovine follicular fluid; (b) determine whether factors shown previously to modulate thecal androgen production (LH, BMPs, EGF, TGFα, TNFα) also modulate thecal *INHA* expression; (c) examine the effects of *INHA* knockdown and exogenous inhibin α subunit treatment on thecal androgen production and (d) test the hypothesis that free inhibin α subunit has an autocrine/paracrine role to modulate thecal androgen production by attenuating the effect of granulosa-derived inhibin (αβ dimer).

## Results

### mRNA expression profiles for INHA and INHBA subunits in developing bovine antral follicles

**Figure 1:**
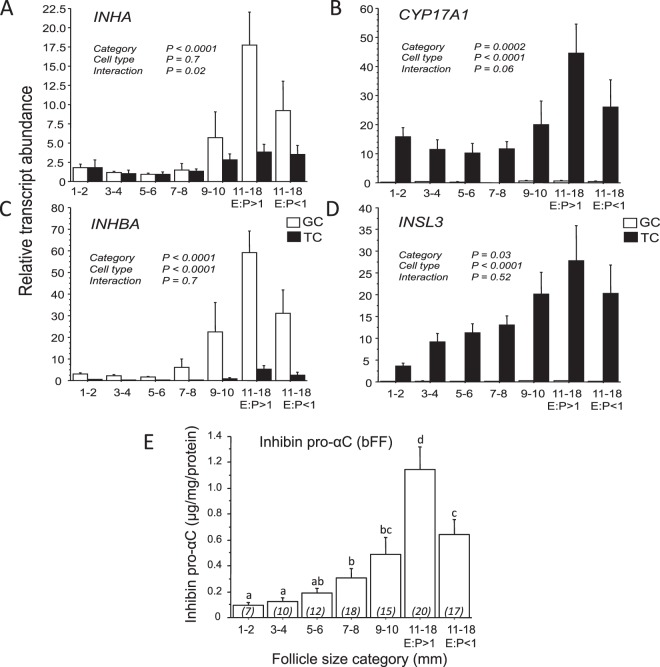
Changes in relative abundance of mRNA transcripts for (**A**) *INHA*, (**B**) *INHBA*, **C**) *CYP17A1* and (**D**) *INSL3* in thecal and granulosal compartments of developing bovine antral follicles. Panel E shows corresponding changes in inhibin α subunit concentration of follicular fluid. Follicles in the 11–18 mm size class have been subdivided on the basis of oestrogen to progesterone ratio (E:P ratio) as ‘estrogen-active’ (E:P ratio >1) or ‘estrogen-inactive’ (E:P ratio <1). Values are means and bars indicate SEM. Results of two-way ANOVA are summarized.

Unexpectedly, appreciable amounts of *INHA* mRNA were detected in TC with levels similar to those in GC found in smaller antral follicles ranging from 1 to 8 mm in diameter (Fig. 1A). The pattern of *INHA* expression in TC was similar to that for *CYP17A1* (Fig. 1B) and *INHA* and *CYP17A1* expression levels positively correlated across the sample set (r = 0.68; p < 0.0001). Expression of *CYP17A1* and *INSL3* mRNA (Fig. 1D) was confined to TC confirming their status as useful TC markers. The relative abundance of *INHBA* transcript (Fig. 1C) was much greater in GC than in TC (P < 0.0001), particularly in large estrogen-active (LEA) follicles that showed greatly increased expression in GC (P < 0.001) compared with GC from all other follicle categories. Likewise, *INHA* mRNA abundance was greatest in GC from LEA follicles (P < 0.01 versus GC from all other follicle categories), consistent with high output of inhibin A (α-βA dimer) by GC of large antral follicles. Concentrations of inhibin α subunit protein measured by ELISA in corresponding bovine follicular fluid samples from these follicles increased ~7-fold between 1–2 and 11–18 mm follicles and followed a similar pattern to those observed for *INHA* and *INHBA* mRNA expression (Fig. 1E).

### Changes in follicular fluid levels of different inhibin α protein forms during antral follicle development

**Figure 2:**
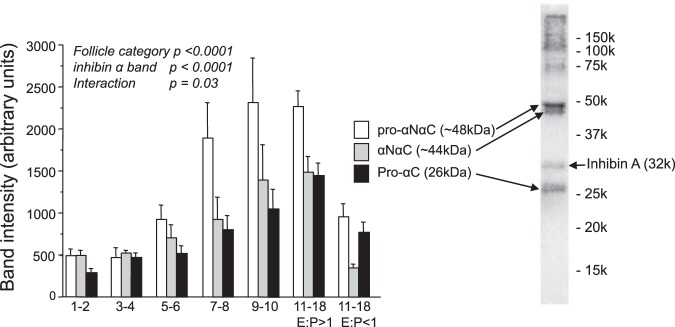
SDS-PAGE/Western blotting analysis (non-reducing conditions) showing changes in the relative abundance of three different molecular mass forms of inhibin α subunit protein follicular fluid from bovine antral follicles. Follicles in the 11–18 mm size class have been subdivided on the basis of oestrogen to progesterone ratio (E:P ratio) as ‘estrogen-active’ (E:P ratio >1) or ‘estrogen-inactive’ (E:P ratio <1). Values are means and bars indicate SEM (n = 6–20 per category). Results of two-way ANOVA are summarized.

Figure 2 shows that the relative amounts of three different molecular mass forms of inhibin α subunit (pro-αNαC, αNαC, pro-αC) in bovine follicular fluid increase in a coordinate manner over the course of antral follicle development, being substantially higher in LEA than in large estrogen-inactive (LEI) follicles. This pattern was comparable to that observed for ‘total’ inhibin α levels determined by pro-αC ELISA (see Fig. 1E).

### Theca cells express immunoreactive inhibin α subunit protein

**Figure 3:**
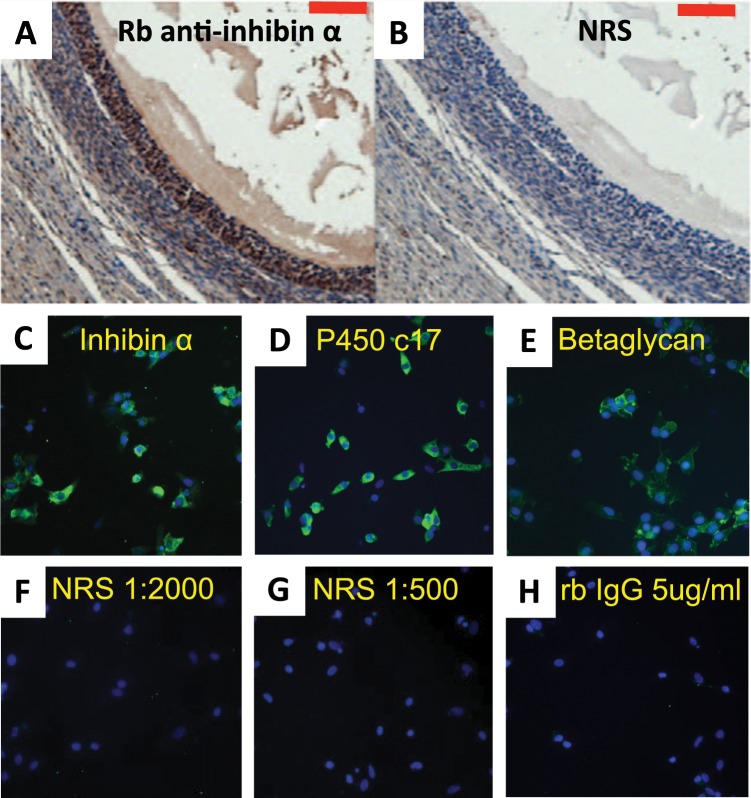
Immunolocalization of inhibin α subunit in (**A**) a bovine antral follicle section and (**C**) cultured bovine theca cells. The latter were also shown to be immunoreactive for (**D**) P450c17 (steroidogenic marker) and (**E**) betaglycan. Antibody controls are shown in panels B, F, G and H. The red bar in A and B = 100 μm; yellow bar in H = 20 μm.

**Figure 4:**
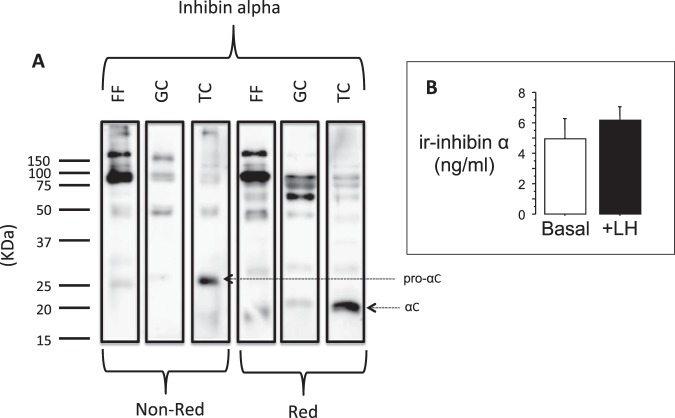
(**A**) Western blotting analysis of inhibin α subunit immunoreactivity in cultured TC-conditioned medium, cultured GC-conditioned medium and pooled bovine follicular fluid, subjected to SDS-PAGE under non-reducing (Non-Red) and reducing (Red) conditions. Note the prominent band (~26 kDa, Non-Red; ~20 kDa, Red) in TC-conditioned medium that corresponds to inhibin pro-αC previously isolated from bovine follicular fluid in the authors’ laboratory (Knight *et al*. 1989). Panel (B) shows that cultured TC secrete immunoreactive pro-αC as detected by 2-site ELISA but that LH stimulation (150 pg/ml) does not affect secretion.

Immunohistochemical staining confirmed the presence of inhibin α subunit protein in the theca interna layer of antral follicles (Fig. 3A). Isolated TC in primary culture were also immunoreactive for inhibin α subunit **(**Fig. 3C**)**, and these cells also expressed P450c17 **(**Fig. 3D**)** and betaglycan protein (Fig. 3E**)**. Immunoblotting analysis of TC-conditioned media (Fig. 4A) revealed a prominent band at ~26 kDa under non-reducing conditions and ~20 kDa under reducing conditions. This indicated that pro-αC is a major form of inhibin α secreted by TC. Indeed, the pro-αC band intensity was much greater than that observed in GC-conditioned media. Higher Mr forms of immunoreactive α subunit were also evident, similar to those observed in GC-conditioned media and bovine follicular fluid. Cultured TC also secreted detectable amounts of free inhibin α subunit measured using 2-site ELISA employing antibodies directed against the pro-region and C-terminal region of the inhibin α subunit presursor (Fig. 4B). Treatment of TC with LH did not affect inhibin α secretion.

### RNAi-mediated knockdown of INHA and INSL3 reduces thecal expression of INHA mRNA and inhibin α subunit protein secretion

**Figure 5:**
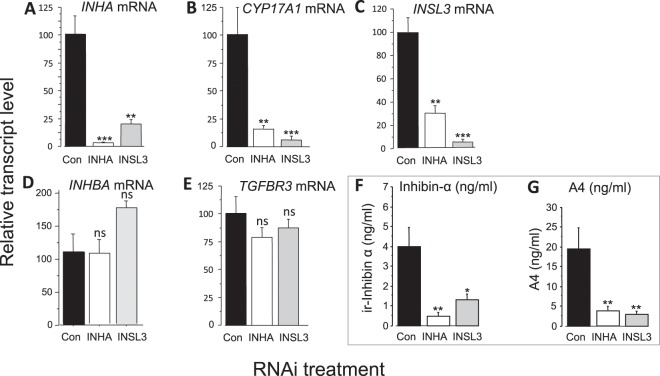
Effect of siRNA-mediated knockdown of *INHA* and *INSL3* on expression of (**A**) *INHA*, (**B**) *CYP17A1*, (**C**) *INSL3*, (**D**) *INHBA* and (**E**) *TGFBR3* mRNA and on secretion of (**F**) inhibin α subunit protein and (**G**) androstenedione (A4) by bovine TCs in vitro. Control cells were transfected with non-silencing control siRNA. Values are means and bars indicate SEM (n = 4 independent cultures). *P     < 0.05, **P < 0.01, ***P < 0.001 vs. control.

Figure 5 shows that in comparison with cells transfected with non-silencing control duplex, transfection with an siRNAi duplex targeting *INHA* reduced *INHA* mRNA level (Fig. 5A) and secreted inhibin α subunit protein level (Fig. 5F) by over 90% (P < 0.01). This was accompanied by an 85% reduction in *CYP17A1* mRNA level (Fig. 5B; P < 0.001) and androstenedione secretion (Fig. 5G; P < 0.01), ~70% reduction in *INSL3* mRNA level (Fig. 5C; P < 0.01) whereas *INHBA* and *TGFBR3* (betaglycan) mRNA level was not affected (Fig. 5DE). Transfection of cells with an siRNAi duplex targeting *INSL3* reduced *INSL3* mRNA level by over 95% (Fig. 5C) and this was accompanied by an ~80% reduction in *INHA* mRNA level (Fig. 5A; P < 0.01) and 70% reduction in secreted inhibin α protein level (Fig. 5F; P < 0.05). *CYP17A1* mRNA level (Fig. 5B; P < 0.001) and androstenedione secretion (Fig. 5G; P < 0.01) were also greatly reduced by *INSL3* knockdown whereas *INHBA* and *TGFBR3* mRNA level was not affected (Fig. 5DE).

### BMPs suppress thecal INHA expression and inhibin α secretion

**Figure 6:**
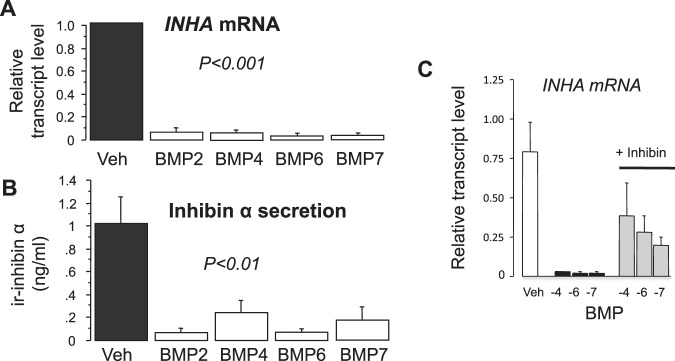
Suppressive effect of BMP 2, -4, -6, and -7 (10 ng/ml) on (**A**) expression of *INHA* mRNA and (**B**) secretion of inhibin α subunit protein by cultured bovine theca cells. The inset (**C**) shows that co-treatment with inhibin-A (50 ng/ml) can partially reverse BMP-induced suppression of thecal *INHA* expression. Values are means and bars indicate SEM (n = 4 independent cultures).

Since BMPs are potent suppressors of thecal *CYP17A1* expression and androgen secretion, we examined the effects of four different BMPs (BMP 2, -4, -6, -7) on *INHA* mRNA expression (Fig. 6A) and inhibin α secretion (Fig. 6B). All four BMPs reduced *INHA* mRNA abundance by over 90% (P < 0.001) while inhibin α secretion was reduced by >80% (P < 0.01). Co-treatment with inhibin reversed the suppressive effect of BMP 4, -6 and -7 on thecal *INHA* expression (Fig. 6C). BMP2 was not included in this experiment.

### Does exogenous pro-αC modulate basal and LH-induced A4 secretion by theca cells?

**Figure 7:**
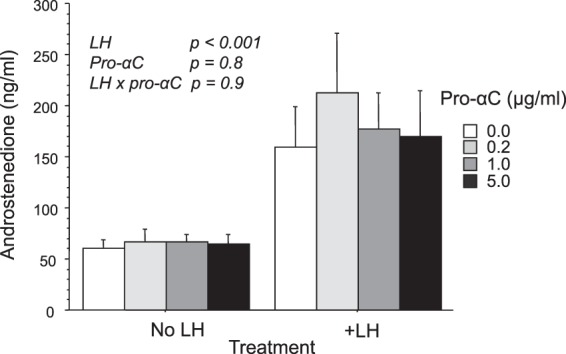
Lack of effect of purified inhibin pro-αC on basal and LH-induced secretion of androstenedione by cultured bovine theca cells. Values are means and bars indicate SEM (n = 4 independent cultures).

Treatment of cultured TC with a range of concentrations (200–5000 ng/ml) of highly purified bovine pro-αC isolated from bovine follicular fluid had no effect on basal or LH-induced androstenedione secretion (Fig. 7)

### Can pro-αC antagonise the effect of inhibin on BMP-treated theca cells?

**Figure 8:**
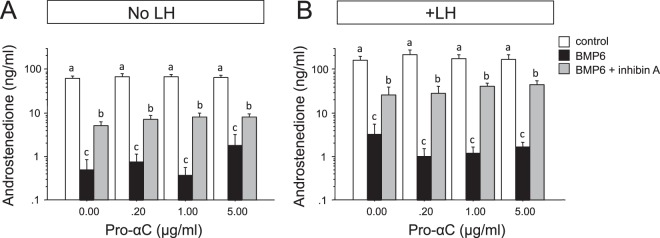
Co-treatment of cultured theca cells with pro-αC does not modulate the ability of inhibin-A to reverse the BMP-induced suppression of thecal androstenedione secretion under either (**A**) basal or (**B**) LH-stimulated conditions. Values are means and bars indicate SEM (n = 4 independent cultures). Within each panel means without a common letter are significantly different (p < 0.05).

Since inhibin has been shown previously to antagonise the suppressive effect of BMP on thecal androgen production^19^, we predicted that exogenous pro-αC would modify this response by impeding inhibin’s action. Cells treated with BMP6 showed a marked reduction in androstenedione secretion under both basal and LH-stimulated conditions (Fig. 8**)**. Co-treatment with purified bovine inhibin-A significantly attenuated the response to BMP6. However, when tested at a range of concentrations (0, 200, 1000, 5000 ng/ml) pro-αC did not modulate the ability of inhibin to reverse the effect of BMP6 on androgen production.

### Do other factors known to modulate thecal steroidogenesis affect INHA expression?

**Figure 9:**
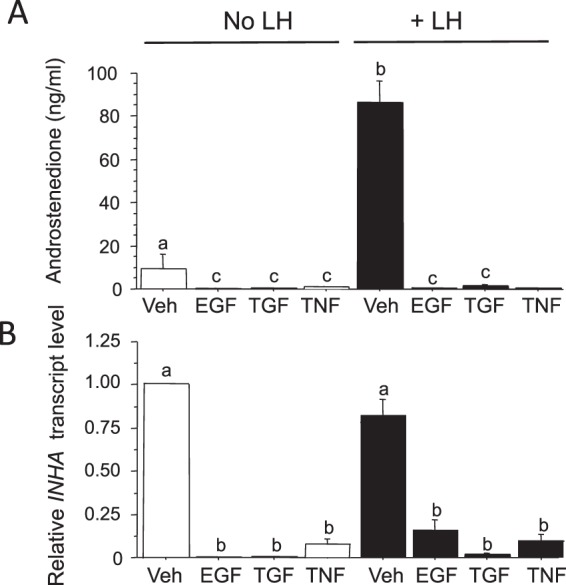
Suppressive effect of EGF, TGFα and TNFα, alone, and in combination with LH, on secretion of androstenedione and expression of *INHA* mRNA by bovine TC. Values are means and bars indicate SEM (n = 4 independent cultures). Means without a common letter are significantly different (P < 0.05).

Figure 9 shows that LH increased androstenedione secretion (p < 0.05) but did not affect INHA expression. Treatment of cells with EGF, TGFα and TNFα greatly reduced both androstenedione secretion (p < 0.05) and *INHA* expression (p < 0.05) under both basal and LH-stimulated conditions.

### Are circulating and intrafollicular androgen concentrations perturbed in heifers actively immunized against inhibin α subunit?

**Figure 10:**
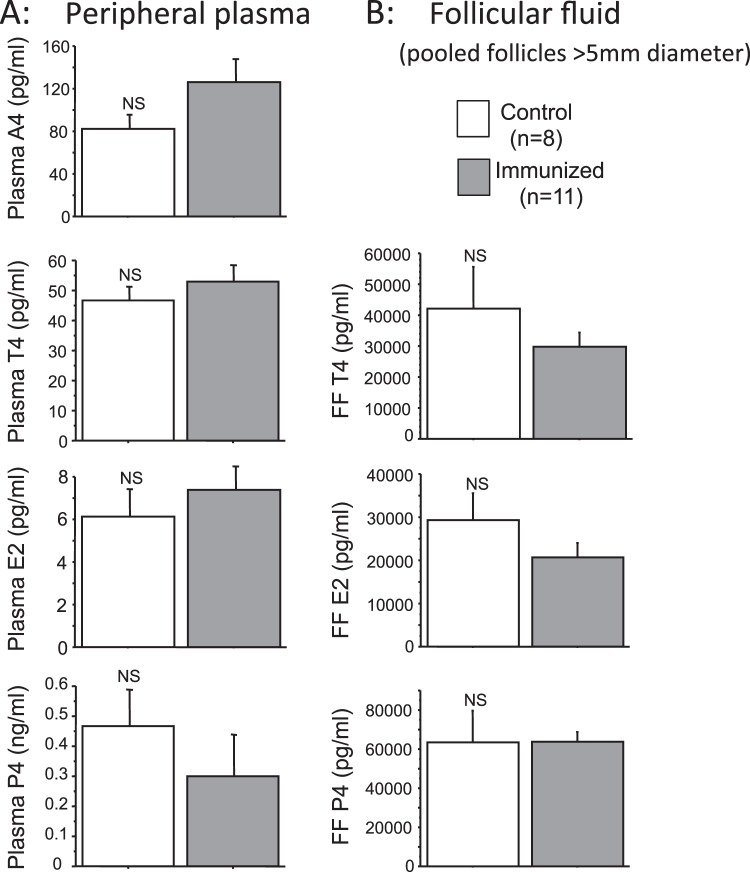
Lack of effect of active immunization against inhibin α subunit on (**A**) circulating and (**B**) intra-follicular steroid concentrations (means ± SEM) in heifers 1–2 days after prostaglandin-induced luteolysis. Androstenedione (A4), testosterone (T4), estradiol (E2) and progesterone (P4).

To test the hypothesis that *in vivo* immunoneutralization of inhibin α subunit in cattle would perturb ovarian androgen output, plasma and intrafollicular steroids were assayed in archived samples from a previous study involving active immunization of heifers against inhibin α subunit^23^. These analyses revealed no significant differences in concentrations of androgen (androstenedione/testosterone), estradiol or progesterone between control and immunized animals sampled during a prostaglandin-synchronized follicular phase (Fig. 10).

## Discussion

‘Free’ inhibin α subunit has previously been detected in, and isolated from, ovarian follicular fluid^10–12^ but its functional significance and cellular origin has received relatively little attention. Here, we report that bovine theca interna cells express substantial amounts of *INHA* mRNA that is translated into inhibin α subunit protein, as evidenced by immunohistochemistry and western blotting of TC-conditioned culture media. Contrary to expectations, relative *INHA* mRNA expression levels in thecal and granulosal layers of small to medium size antral follicles were found to be similar. Different M*r* forms of inhibin α subunit were secreted by cultured TC including pro-αC, previously identified in and isolated from bovine FF in this laboratory^10^. Taken together, these observations suggest that the theca interna layer may contribute significantly to the inhibin α subunit content of antral follicular fluid, hitherto considered to be primarily of granulosal origin^4,5,24^. Similarly, inhibin α subunit present in utero-ovarian vein and jugular vein plasma of cattle^10^ may be partially of theca origin.

The follicular gene expression profile we observed in GC accords with the consensus view^4,25–27^ that *INHBA* (encoding inhibin/activin βA subunit precursor) is predominantly expressed by GC of growing follicles with maximal expression observed in large oestrogen-active follicles, concomitantly with maximal expression of *INHA* (encoding inhibin α subunit precursor), thus supporting the capacity for high inhibin αβ dimer production by GC. Nonetheless, some *INHBA* expression was also evident in the TC layer of large (11–18 mm) follicles raising the possibility that TC in these follicles contribute to the follicular output of inhibin. Indeed, low but detectable amounts of inhibin A were detected in TC-conditioned media, with levels 13–30 fold less than in GC-conditioned media (see Supplementary Fig. 1). In contrast, activin A levels were undetectable in these TC-conditioned media. Moreover, the observation that both follistatin and inhibin A were capable of reversing activin A-induced suppression of thecal androgen secretion, but did not raise androgen production when added alone, further supports a lack of endogenous activin production by bovine TC (see Supplementary Fig. 2). Thecal expression of *INHBA*, *INHBB* and *INHA* mRNA has also been reported in sheep ovarian follicles^28^. In the present study, while thecal *INHA* expression level only increased ~2 fold in follicles >9–10 mm in diameter, granulosal expression level increased by ~10-fold to peak in LEA follicles. Taken together, the above observations suggest that while GC are capable of producing functional dimeric inhibins and activins, ‘free’ inhibin α subunit is mostly produced by the follicular TC layer.

This was supported, at least in part, by a western blotting comparison of pooled TC- and GC-conditioned culture medium with pooled bovine follicular fluid. This revealed a prominent ~26 kDa band of immunoreactive inhibin α subunit in TC-conditioned media that migrated with an apparent Mr of ~20 kDa under reducing conditions. This band corresponds to the inhibin pro-αC form previously isolated from bovine follicular fluid^10–12^ and its greater abundance in TC-conditioned medium than in GC-conditioned medium was unexpected. Other higher M*r* immunoreactive bands were present in all three samples reflecting differential processing of the inhibin subunit precursors. The ability of cultured TC to secrete inhibin pro-αC was directly confirmed by 2-site ELISA although it should be cautioned that this assay would also be expected to cross react with other molecular forms (including α-β dimeric inhibins) that contain both pro- and carboxy-terminal regions of the α subunit precursor. Expression of *INHA* mRNA and inhibin α protein has also been reported in the theca layer of human antral follicles^29^ but we are not aware of any studies to explore the functional significance of thecal inhibin α in the human ovary.

We examined the effects on thecal *INHA* expression of several factors shown previously to enhance (LH) or suppress (BMPs, TNFα, TGFα and EGF) androgen production by cultured TC^30–32^. Whilst LH, the primary endocrine signal promoting thecal androgen production, had no effect on INHA expression, the finding that each of the above intra-ovarian growth factors concomitantly suppressed *INHA* expression and androgen secretion provides further, albeit indirect, evidence that inhibin α subunit may contribute to the regulation of thecal steroidogenesis.

Several biological activities have been ascribed to ‘free’ inhibin α subunit including inhibition of FSH binding to its receptor^13^ and inhibition of oocyte maturation^14^. In addition, immunization of ewes against the αN fragment of the inhibin α subunit precursor was associated with an increased incidence of unruptured ovulatory follicles and reduced fertility^33^. As discussed below, free inhibin α subunit may also function as an inhibin antagonist by competing with inhibin for binding to betaglycan.

Betaglycan acts as a co-receptor for inhibin, to which it binds via its α subunit. This association greatly enhances presentation of inhibin to type 2 signaling receptors on the cell surface and facilitates its antagonism of activin signalling by preventing formation of signalling complexes between type 2 and type 1 receptors^18^. In the same betaglycan-dependent manner, inhibin can antagonise the effects of BMPs^19–21^. We reported previously that thecal betaglycan mRNA expression increases progressively during bovine antral follicle growth from 1–10 mm, preceding the major rise in inhibin α/β subunit expression evident in GC of follicles >9–10 mm in diameter^19^. This led to the proposal that the ability of GC-derived inhibin to interact with neighbouring TC depends on this upregulation of betaglycan expression in growing follicles. In this context, intrafollicular BMPs potently suppress LH-induced androgen production^30^, and this effect is reversed by inhibin^19^. Thus, enhanced TC androgen output, required for the subsequent conversion to oestrogens by GC as follicles progress towards functional dominance is likely to depend, at least in part, on increased GC inhibin production. Based on the ability of inhibin to bind betaglycan through its α subunit^18,20,22^, we tested the hypothesis that ‘free’ inhibin α subunit synthesized by theca cells might function as an intrafollicular inhibin antagonist (or BMP agonist) by reducing presentation of inhibin to type-2 BMP/activin signalling receptors on the theca cell surface. However, we show here that co-treatment of theca cells with exogenous inhibin α subunit isolated from bovine follicular fluid did not modify the ability of purified bovine inhibin-A to reverse the BMP-induced suppression of androstenedione secretion, casting doubt on the validity of this hypothesis. Whilst it is possible that the inhibin α subunit preparation had lost its biological activity during the isolation process, this seems unlikely given that the inhibin-A preparation (isolated in parallel using the same chromatographic techniques) was biologically active.

Interestingly, BMP-induced suppression of *CYP17A1* expression and androgen secretion was accompanied by a marked suppression of thecal *INHA* mRNA expression and inhibin α subunit protein secretion raising the possibility of a positive role for inhibin α in the maintenance of thecal androgen production. Indeed, such a role was supported by our observation that RNAi-mediated knockdown of *INHA* decreased thecal *CYP17A1* expression and androgen synthesis. Correspondingly, we anticipated that treating cells with exogenous inhibin α subunit would raise androgen production, particularly in BMP6-treated cells with greatly reduced *INHA* expression. However, no effect of inhibin α subunit was found under basal or LH-stimulated conditions (see Fig. 7), or in BMP6-treated cells (see Fig. 8) suggesting a more complex (indirect) relationship between BMP signalling, *INHA* expression and androgen biosynthesis. A recent study involving a mouse Leydig cell line (TM3) also showed that *INHA* knockdown decreased expression of *cyp17a1* and several other transcripts including *Insl3*^34^. Moreover, TGFβ- and activin-induced Smad2 activation was enhanced by *INHA* knockdown in TM3 cells. This was interpreted as evidence that endogenous inhibins, whose synthesis would be compromised by *INHA* knockdown, counteract the negative effects of TGFβ and activin on TM3 cells.

Notably, *INSL3* expression was also greatly reduced by *INHA* knockdown in TC suggesting a possible association with INSL3-RXFP2 signaling recently shown to contribute, along with BMPs, to the regulation of thecal androgen production in the bovine^31,35^. As mentioned above *INHA* knockdown also led to a reduction in *insl3* expression in mouse TM3 cells^34^. Indeed the present finding that BMP 2, 4, 6 and 7 each suppressed *INHA* expression and inhibin α protein secretion confirms and extends our previous microarray data showing a substantive reduction of thecal *INHA* expression in BMP6-treated theca cells, with *INHA* being the third most heavily suppressed transcript in that study^31^. Conversely, it was also found that knockdown of *INSL3* greatly reduced *INHA* mRNA level and inhibin α protein secretion, concomitantly with reduced *CYP17A1* expression and androgen secretion. This suggests a reciprocal stimulatory action of thecal inhibin α subunit and *INSL3* on each other’s expression, and on androgen production.

The present findings also raise questions about the mode of action of inhibin αC subunit auto-immunization, a vaccination procedure that has been found to enhance ovarian follicle development and raise ovulation rate in several species including cattle^36,37^, sheep^38–40^ and goat^41,42^. Previously, it was considered that this action primarily involved neutralization of inhibin negative feedback action on pituitary FSH secretion, resulting in raised FSH levels that, in turn, enhance follicle development. However, based on the present *in vitro* findings, we considered the possibility that inhibin α antibodies may also directly target follicular theca cells, thereby disrupting intrafollicular autocrine/paracrine action(s) of theca-derived inhibin α subunit. However, when we examined this possibility by analysing intrafollicular and circulating androgen concentrations in heifers actively immunized against inhibin α subunit, no significant differences were observed in comparison with non-immunized controls, thus casting doubt on this suggestion.

**Figure 11:**
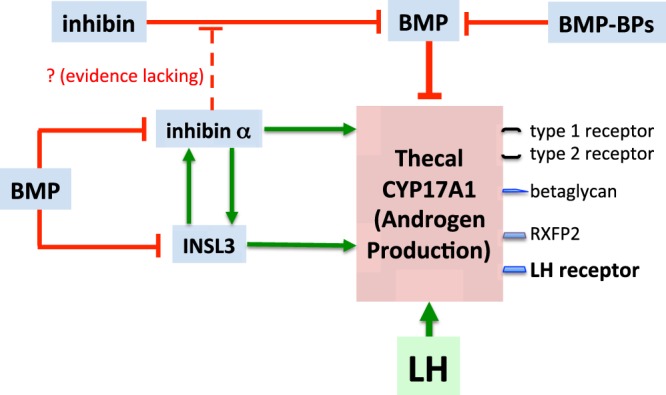
Schematic diagram depicting proposed intrafollicular interactions between BMPs, inhibin, BMP-binding proteins (BPs), inhibin α subunit and INSL3 that contribute to the regulation of *CYP17A1* expression and androgen production by theca cells.

In conclusion, following on from the finding that thecal betaglycan (inhibin co-receptor) and INSL3 expression increase progressively throughout follicle growth^19,35^, here we report that TC also express substantial amounts of *INHA* mRNA and ‘free’ inhibin α subunit protein. Moreover, knockdown of thecal *INHA* suppressed *CYP17A1* expression and androgen synthesis. Betaglycan and inhibin α have the potential to modulate the action of BMPs and inhibin on TC. BMPs concomitantly suppressed *INSL3*, *INHA*, *CYP17A1* mRNA expression and secretion of INSL3, inhibin α subunit and androgen, actions that were reversed by inhibin A. However, no *in vitro* evidence was forthcoming to support the hypothesis that free inhibin α subunit antagonises the effect of inhibin on BMP-induced attenuation of thecal androgen production. Furthermore, *in vivo* immunoneutralization of inhibin α did not perturb ovarian androgen production in cattle. In the light of these inconclusive findings, additional studies are needed to define the role(s) of thecal inhibin α subunit and to unravel the specific interactions of inhibin, ‘free’ inhibin α subunit, betaglycan, INSL3 and BMPs in the context of ovarian follicle steroidogenesis. Figure 11 is a schematic diagram depicting proposed interactions envisioned for BMPs, inhibin, BMP-BPs, inhibin α subunit and INSL3 that may contribute to the regulation of *CYP17A1* expression and androgen production by theca cells.

## Materials and Methods

### Preparation of granulosal (GC) and theca interna (TC) extracts for follicular gene expression profiling

Antral follicles ranging in diameter from 1 to 18 mm were dissected from ovaries of cattle obtained from an abattoir as described previously^19^. Follicles were sorted by size and their GC, TC layers and follicular fluid were recovered. Isolated GC and TC were homogenised in 0.5 ml of Tri-reagent (Sigma UK Ltd) and stored at −80 °C prior to RNA extraction. A total of 83 TC samples and 88 GC samples were included in the present analysis approximately half of which were generated in the above-mentioned study^19^. Concentrations of estradiol-17β and progesterone in follicular fluid (n = 99 samples) were determined by immunoassay while total protein concentration was determined by coomassie blue dye-binding assay with bovine serum albumen as the calibration standard. Follicles in the largest size category (11–18 mm) were subdivided according to their follicular fluid estradiol:progesterone (E:P) ratio. Those with an E:P ratio >1 were presumed to be healthy, ‘estrogen-active’ dominant follicles, hereafter referred to as large estrogen-active (LEA) follicles. Follicles with an E:P ratio <1 were presumed to be ‘estrogen inactive’ and regressing and are referred to as large estrogen-inactive (LEI) follicles.

### Collection of follicular fluid for western blotting analysis of inhibin α subunit forms

A separate set of follicular fluid samples (n = 84) was collected as above for SDS-PAGE/western blotting analysis of different molecular weight forms of inhibin α subunit over the course of antral follicle development. Estradiol-17β and progesterone concentrations were also measured to allow classification of 11–18 mm follicles into LEA and LEI categories on the basis of E:P ratio.

### Primary theca interna cell culture

TC were isolated from bovine ovaries obtained from the slaughterhouse as described previously^30,31^. Pooled TC from approximately 50 individual 4–6 mm follicles were seeded into either 96-well plates (7.5 × 10^4^ viable cells/well) for evaluation of hormone secretion by immunoassay and viable cell number by neutral red uptake assay, or in 24-well plates (0.5 × 10^6^ viable cells/well) for RNA extraction and gene expression analysis. Cells were routinely cultured for 6 days under defined serum-free conditions with treatments present on days 3–6 inclusive. For RNAi knockdown experiments cells were cultured for 7 days in total. The culture medium used was McCoy’s 5 A supplemented with 1% (v/v) antibiotic-antimycotic solution, 10 ng/ml bovine insulin, 2 mM L-glutamine, 10 mM HEPES, 5 µg/ml apo-transferrin, 5 ng/ml sodium selenite and 0.1% (w/v) BSA (all purchased from Sigma UK Ltd, Poole, UK). Antibiotic-antimycotic was omitted from the culture medium used during transfection of cells with small interfering (si)RNA duplexes in accordance with the protocol provided by the supplier. Media were changed on days 3 and 5 with fresh media containing treatments as appropriate. Conditioned media were collected at the end of culture for immunoassay and cell lysates were prepared using RNeasy lysis buffer (Qiagen). Pooled lysates from replicate wells were stored at −80C until total RNA isolation.

### Cell culture treatments

Highly purified ovine LH (NIADDK oLH-S-16) was obtained from the National Hormone and Pituitary Program (NHPP), Torrance, CA, USA. Recombinant human (rh) BMP2, BMP4, BMP6, BMP7, TNFα, TGFα and EGF were purchased from R&D systems (Abingdon, UK). Highly purified bovine inhibin A and pro-αC were prepared ‘in house’ from pooled bovine follicular fluid (see below). Treatment solutions were sterilized using 0.2 µm membrane filters before dilution in sterile culture medium to required concentrations. For experiments involving knockdown of endogenous *INHA* and *INSL3*, siRNA duplexes against bovine *INHA* (sense strand: GGGAACUUGUCCUGGCCAAUU; antisense strand: UUGGCCAGGACAAGUUCCCUU) and bovine *INSL3* (Sense strand: GGCAAGACCUGCUGACCCUUU; antisense strand: AGGGUCAGCAGGUCUUGCCUU) were custom-designed and synthesized by Dharmacon Thermo Scientific (Lafayette, CO, USA). Controls included cells transfected with a non-silencing control RNAi (NSC3; Dharmacon) as well as cells exposed to transfection reagent only (DharmaFECT 2; Dharmacon). All cell culture experiments were repeated using TC prepared from n = 4 independent batches of follicles.

### Theca cell culture experiments 1–5

#### Effects of RNAi-mediated knockdown of INHA and INSL3 on thecal expression of INHA mRNA and inhibin a subunit protein secretion

To investigate the effect of knockdown of endogenous *INHA* and *INSL3*, TC were transfected with siRNA duplexes (50 nM final concentration), non-silencing control duplex (NSC3; Dharmacon; 50 nM) or transfection reagent only (DharmaFECT 2; 0.25 μl/well). siRNA duplexes were prepared for transfection as recommended in the supplier’s protocol (Dharmacon) and added to cells, together with LH (150 pg/ml), after medium changes on days 2 and 4 of culture. Cells were lysed 3 days later for RNA extraction and RT-qPCR analysis while media were retained for androstenedione and inhibin α subunit assay.

#### Effect of BMPs on thecal INHA expression and inhibin α secretion

Theca cells cultured in 24-well plates were treated for 4 days with BMP2, 4, 6 and 7 (each at 10 ng/ml). In addition, the effects of co-treatment with inhibin A (50 ng/ml) on the responses to BMP 4, 6 and 7 were also examined. Expression of *INHA* mRNA was analysed by RT-qPCR and concentrations of inhibin α subunit protein in conditioned media determined by 2-site ELISA.

#### Does exogenous pro-αC modulate basal and LH-induced androstenedione secretion?

TC cultured in the presence and absence of LH (150 pg/ml) were treated for 4 days with highly purified bovine inhibin pro-αC (0, 200, 1000 and 5000 ng/ml). Media were changed and treatments replenished after 2 days. Concentrations of androstenedione in conditioned media were determined by ELISA.

#### Does exogenous pro-αC modulate the ability of inhibin to reverse the BMP-induced suppression of thecal androgen secretion?

Since inhibin has been shown previously to antagonise the suppressive effect of BMP on thecal androgen production^19^, we evaluated whether exogenous inhibin pro-αC could modify this response. Under both ‘basal’ and LH-stimulated conditions TC were treated with/without BMP6 (10 ng/ml) in the presence and absence of purified bovine inhibin-A (500 ng/ml). The above treatment matrix was replicated in cells co-treated with purified inhibin pro-αC at three different concentrations (200, 1000, 5000 ng/ml). At the end of culture, androstenedione concentrations in conditioned media were determined by ELISA.

#### Do other factors known to modulate thecal steroidogenesis affect INHA expression?

Since TNFα, TGFα and EGF have been shown previously to suppress thecal androgen production^31,32^, we examined the effects of these peptides (10 ng/ml) on *INHA* expression by TC cultured in the absence and presence of LH (150 pg/ml).

### Are circulating and intrafollicular androgen concentrations perturbed in heifers actively immunized against inhibin α subunit?

To test the hypothesis that *in vivo* immunoneutralization of inhibin α subunit would perturb ovarian androgen output and circulating androgen concentrations, we measured androstenedione, testosterone, estradiol and progesterone concentrations in peripheral plasma samples collected during a prostaglandin (PG) synchronized follicular phase from control (n = 8) and inhibin α-immunized (n = 11) heifers. Ovaries were recovered after slaughter (day 2 after PG administration) and mean steroid concentrations determined in follicular fluid samples pooled from all follicles >5 mm in diameter. These samples had been collected during a previous study focussing on the effect of inhibin α immunization on ovulation rate^23^. The study was conducted in full accordance with the UK Animals (Scientific Procedures) Act (1986). Mean inhibin α antibody titres (1:2000 plasma dilution) in control and immunized heifers were 0.8 ± 0.06 and 20.7 ± 4.5% respectively.

### SDS-PAGE and Western Blotting

Before SDS-PAGE, GC- and TC-conditioned cell culture media samples were concentrated ~10-fold using centrifugal 10 kDa cut-off membrane filter devices (Amicon Ultra, Amicon). Buffer exchange was achieved by loading the filtration devices with non-reducing SDS-PAGE sample buffer before centrifugation. Follicular fluid samples and concentrated media samples (50 μg protein) were electrophoresed under non-reducing and/or reducing conditions using 12.5% gels. After semi-dry transfer to nitrocellulose membranes, immunodetection was performed in one of two ways. For semi-quantitative analysis of different MW forms of inhibin α in bovine follicular fluid, membranes were incubated overnight with monoclonal antibody against inhibin α subunit (clone PPG14; 1 μg/ml; Prof NP Groome, Oxford Brookes University). After washing, ^125^I-labelled goat anti-mouse IgG (10^5^ cpm/ml 3 h incubation at room temperature) was used for signal detection and images were captured using a phosphor screen and phosphorimager (Molecular Dynamics) with subsequent analysis using Image J 1.32^43^. For additional experiments comparing cell-conditioned media and bovine follicular fluid samples, a biotinylated form of the same primary antibody (PPG14; 0.5 μg/ml) was used for immunodetection with signal detection achieved using an ABC peroxidase kit (Vector Laboratories, Peterborough, UK) followed by chemiluminescent substrate (ECL reagent, GE Healthcare) and film-based image capture.

### Immunohistochemistry

Immunohistochemistry was performed on formalin-fixed paraffin embedded cow ovaries as described previously^35^. After quenching endogenous peroxidase using hydrogen peroxide, microwave antigen retrieval (10 mM citrate buffer, pH 6) was carried out and sections were blocked using 2.5% (v/v) horse serum. Sections were incubated overnight (4 °C) with an ‘in house’ rabbit antibody raised against residues 1–29 of the αC subunit of bovine inhibin (PPD2/4; 1/2000 dilution). Control sections were incubated with normal rabbit serum (1/2000). After washing slides in PBS/Tween the ImmPRESS Universal anti-mouse/rabbit IgG HRP polymer-based detection system (Vector Laboratories) was used with diaminobenzidine substrate according to the manufacturer’s instructions. Sections were counterstained with haematoxylin, dehydrated and mounted. For immunocytochemistry, TC were cultured in NUNC chamber slides and fixed for 30 min in 4% paraformaldehyde in PBS (pH 7.4). After permeabilization using 0.1%(v/v) Triton X-100 in PBS (10 min), cells were washed in PBS (2 × 5 min) and blocked (1 h) in PBS containing 0.1% NaN3, 2% BSA and 10% normal goat serum. After overnight incubation at 4 °C with rabbit anti-inhibin α subunit (PPD2/4; 1/2000), rabbit anti-betaglycan (Santa Cruz sc-6199; 5 μg/ml), or rabbit anti-P450 c17 (Gift from Professor S. Kominami, Hiroshima University, Hiroshima, Japan; 1:500) or with equivalent concentrations of normal rabbit or mouse IgG as controls, slides were washed (3 × 10 min in PBS) and incubated for 1 h with fluor-labelled secondary antibody (10 μg/ml; goat anti-rabbit IgG-FITC conjugate). After washing in 0.1% Triton X-100 in PBS for 1 h and then PBS (3 × 10 min) slides were mounted in DAPI-contaning medium (Vectashield, Vector Laboratories). Images were captured on a Zeiss Axioskop 2 microscope using a Zeiss Axiocam camera with Axiovision software.

### RT-qPCR

cDNA was synthesized from 1 µg of total RNA using the AB high capacity cDNA reverse transcription kit used according to the manufacturer’s instructions (AB, Life Technologies, Paisley, UK). Primers (Table 1) were designed using Primer Express software (AB) or Primer-Blast (www.ncbi.nlm.nih.gov/tools/primer-blast/). PCR assays were carried out in a volume of 14 µl, comprising 5 µl cDNA (1/50 dilution), 1 µl each forward and reverse primers (final concentration 360 nM) and 7 µl SYBR Green 2x Master Mix (QuantiTect; Qiagen, UK). Real-time PCR assays were run using an AB StepOne Plus instrument (Life Technologies, Paisley, UK) as described elsewhere^19^. The ΔΔCt method^44^ was used for comparison of relative transcript abundance. Transcript Ct values for each sample were first normalized to *ACTB* Ct value (which was uniform across all experimental groups: ANOVA *P* > 0.1) to generate ΔCt values. In the case of the follicular GC and TC samples set ΔCt values for each transcript in a given sample were then normalized to the mean ΔCt value for that transcript in all tissue samples. In the case of cell culture experiments ΔCt values for individual replicates within each treatment group were normalized to the mean ΔCt value of vehicle-treated controls. For graphical presentation of results ΔΔCt values were finally converted to fold-differences using the formula: fold-difference = 2^(−∆∆Ct).^

**Table 1:** Primers used for real-time PCR.

Target	Accession number	Forward primer 5′ to 3′	Reverse primer 5′ to 3′
INHBA	NM_174363.1	GAAGAGACCCGATGTCACCCAGC	TGTCGTCCTCTATCTCCACGTACCCG
INHA	NM_174094.3	GAGCCCGAGGACCAAGATGTCTCC	CCTCAGCCTCTCCAGCATCTGGC
TGFBR3	XM_001253071.2	TGCACTTTCCTATCCCACAAGCCG	CCAGATCATTGAGGCATCCAGCG
CYP17A1	NM_174304	GACAAAGGCACAGACGTTGTGGTCA	TGATCTGCAAGACGAGACTGGCATG
INSL3	NM_174365.2	TCTGTCCCCACTGAATCCTCCTGG	GGGTTTCATGGTGCTGTGTGGC
ACTB	BC102948.1	ATCCACCATCGGCAATGAGCGGTT	CGGATGTCGACGTCACACACTTCATG

### Steroid immunoassays

Steroid concentrations in cell culture media, follicular fluid and plasma samples were determined by competitive immunoassays as described previously^30,31,35,36^. Mean within- and between-assay CVs were <8% and <12%, respectively for all analytes.

### Two-site immunoassay for inhibin pro-αC

Concentrations of inhibin pro-αC in TC-conditioned media were determined using a modified version of a previously reported 2-site ELISA^45^ in which the alkaline phosphatase-labelled antibody (anti human αC region) was substituted with a monoclonal antibody (PPG14) directed against residues 1–32 of the αC fragment of the bovine inhibin α subunit precursor, to make the assay more applicable to the bovine species. In addition, the assay was performed in solid black microplates and a fluorogenic substrate (4-methylumbelliferyl phosphate) was used instead of a colorimetric substrate to quantify the amount of bound alkaline phosphatase. Fluorescence signals were captured using a multi-function microplate reader (Fluostar Optima; BMG Labtech, Aylesbury, Bucks, UK) with excitation and emission wavelengths of 364 nm and 448 nm, respectively. Highly purified bovine pro-αC purified in this laboratory was used as the assay standard. The detection limit was 30 pg/ml and mean intra- and inter-assay CVs were 7% and 10% respectively.

### Purification of bovine inhibin A and inhibin pro-αC

Inhibin A and inhibin pro-αC were isolated from pooled bovine follicular fluid as described previously^14^ but with some modifications. Briefly, frozen bovine follicular fluid (65 mg protein/ml) was thawed, centrifuged (20,000 g, 30 min, 4 °C) and the supernatant (150 ml; 9.75 g protein) subjected to an immunoaffinity extraction step. The immunoaffinity matrix was prepared by coupling 10 mg of a monoclonal antibody raised against bovine αC (clone PPG14) to 5 ml of n-hydroxysuccinamide (NHS)-activated sepharose beads (GE Healthcare Ltd), according to the manufacturer’s instructions. Follicular fluid was mixed with the immunoaffinity beads overnight at 4 °C using a rotating mixer. The suspension was then transferred to an empty column (10 × 80 mm), fitted with a porous polyethylene frit) and the retained beads washed by passing through 150 ml PBS followed by a final ‘high salt’ wash (50 ml) of 0.5 M sodium acetate. The antibody-bound fraction was then eluted from the beads using 15 ml of 8 M urea solution. After immunoaffinity extraction, reversed phase HPLC was used to resolve fractions containing 32 kDa inhibin A and ~26 kDa inhibin pro-αC using a TSK-ODS-120T C18 (4.6 × 250 mm) column (Hichrom Ltd, Berks, UK). The column was developed with a 5 min linear gradient of 10–25% acetonitrile in 0.1% trifluoroacetic acid (TFA), followed by a 40 min gradient of 25–75% acetonitrile at a flow rate of 1 ml/min with fraction collected every 0.5 min. Highly enriched inhibin A and pro-αC-containing fractions from 12 runs were pooled and each pooled sample was re-applied (2 runs each) to the same RP-HPLC column that was developed with the same gradient at 1 ml/min; fractions were collected every 0.25 min. HPLC column eluates were monitored for UV absorbance at 280 nm and fractions were analysed using two-site immunoassay for inhibin A^46^ and pro-αC (see above). Estimates of total protein concentrations and final yields were based on the assumption that a 1 mg/ml solution of protein has a 280 nm absorbance value of 1.0. ELISA revealed that the final pro-αC-containing RP-HPLC preparation contained significant (7%) contamination with inhibin A (α-βA dimer). Therefore, an additional immunoaffinity step was employed in which the sample was lyophylized, redissolved in PBS and passed through a column containing a 1 ml bed of NHS-activated sepharose beads coupled to 1 mg anti-βA mAb (clone E4, Groome). This step effectively depleted the inhibin A content of the pro-αC preparation to 0.26%. Table 2 summarized the purification scheme used to isolate inhibin A and pro-αC from bovine follicular fluid and the resultant yields.

**Table 2:** Summary of purification scheme used to isolate inhibin-A and pro-αC from bovine follicular fluid (bFF).

Step	Fraction	Volume (ml)	inhibin-A (μg)	Inhibin-A yield (%)	pro-αC (μg)	pro-αC yield (%)
1	bFF starting material (9750 mg total protein)	150	1888	100	2995	100
2	PPG mAb affinity beads (8 M urea eluate)	30	443	23.4	1665	55.6
3	RP-HPLC (fractions 41–44)	60 (4 × 15 runs)	**195**	**10**.**3**	<DL	na
4	RP-HPLC (fractions 48–50)	45 (3 × 15 runs)	59	3.2	840	28.5
5*	E4 mAb affinity beads (non-retained fraction)	45	1.8	0.1	**701**	**23**.**4**

Values in bold indicate the final preparations (step 3: inhibin A; step 5: pro-αC) that were freeze dried as 5 or 10 μg aliquots and stored at −80C. Note that step 5 was only applied to pro-αC fraction from step 4 to reduce residual inhibin-A contamination from 7.04% to 0.26%.

### Statistical analysis

*In vitro* hormone secretion data were log-transformed prior to statistical analysis using using one- or two-way ANOVA. Where indicated, Fisher’s PLSD test was used for *post-hoc* pairwise comparisons. Values are presented as arithmetic means ± SEM based on 4 independent culture experiments. RT-qPCR data from 4 independent batches of cells were analysed (ANOVA and *post-hoc* Fisher’s PLSD test) as ΔCt values (i.e. log_2_ values) before conversion to fold-difference values for graphical presentation as arithmetic means ± SEM. Steroid hormone concentrations in plasma and follicular fluid from control and inhibin-immunized cattle were compared by Student’s unpaired t-test.

## Supplementary information

Supplementary Information

## Data Availability

The datasets generated during and/or analysed during the current study are available from the corresponding author on reasonable request.
