# Supplementary material for: ‘Free’ inhibin α subunit is expressed by bovine ovarian theca cells and its knockdown suppresses androgen production
Source: Sci Rep. 2019 Dec 24;9:19793. doi: 10.1038/s41598-019-55829-w (PMC6930203; doi:10.1038/s41598-019-55829-w)

Supplementary Information with MS: 'Free' inhibin α subunit is expressed by bovine ovarian theca cells and its knockdown supresses androgen productionM Laird, C Glister, W Cheewasopit, LS Satchell, AB Bicknell, PG Knight

Supplementary Figure 1 Comparison of (A) inhibin A and (B) activin A secretion by cultured granulosa (GC) and theca (TC) cells under basal and gonadotrophin-stimulated conditions. Inhibin A levels in TC-conditioned media were substantially lower than in GC-conditioned media. Activin A levels in TC-conditioned media were below the detection limit of the assay* (~100pg/ml). Values are means and bars indicate SEM (n=4 independent cultures). Means without a common letter are significantly different (P<0.05). *Knight PG, Muttukrishna S & Groome NP (1996) Development and application of a two-site enzyme immunoassay for the determination of 'total' activin-A concentrations in serum and follicular fluid. J Endocrinol. 148, 267-279

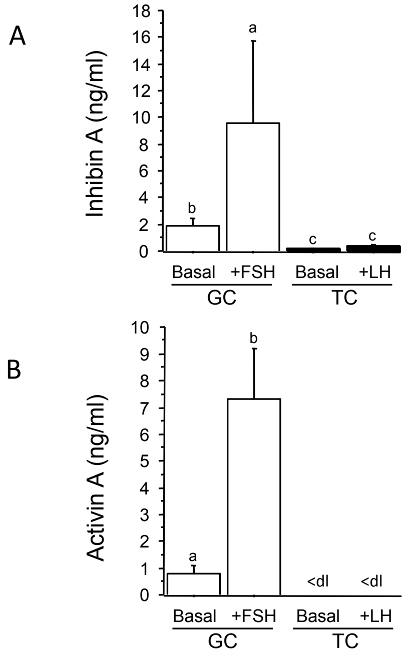

Supplementary Figure 2 Both (A) inhibin A and (B) follistatin reverse the suppressive effect of activin A (10 ng/ml) on LH-induced androstenedione secretion but neither peptide affects androstenedione secretion in the absence of exogenous activin A suggesting a lack of endogenous activin production by theca cells. Values are means and bars indicate SEM (n=4 independent cultures). Different letters above bars indicate significant differences (P<0.05)

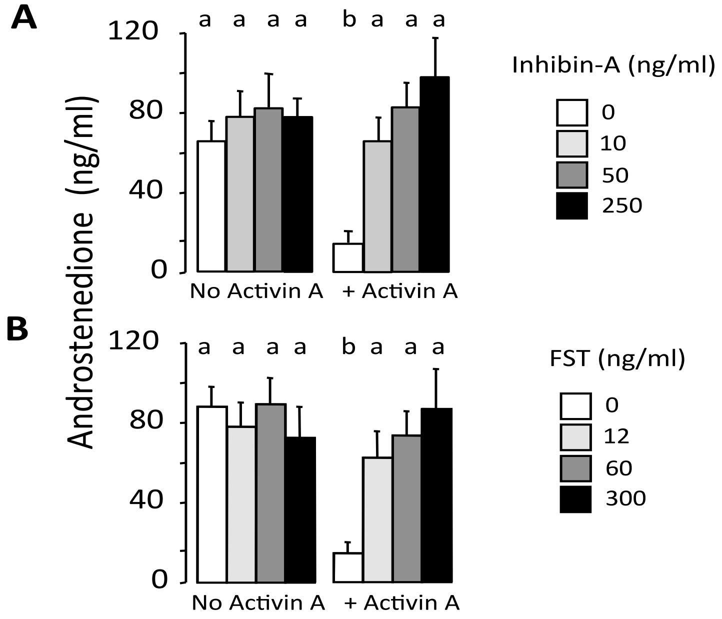

Supplement: Supplementary file 1 — Supplementary Information [file 41598_2019_55829_MOESM1_ESM.pdf]
